# Excess protein enabled dog domestication during severe Ice Age winters

**DOI:** 10.1038/s41598-020-78214-4

**Published:** 2021-01-07

**Authors:** Maria Lahtinen, David Clinnick, Kristiina Mannermaa, J. Sakari Salonen, Suvi Viranta

**Affiliations:** 1 Finnish Food Authority, Mustialankatu 3, 00790 Helsinki, Finland; 2 Laboratory of Chronology, Finnish Museum of Natural History, University of Helsinki, PO Box 64, 00014 Helsinki, Finland; 3 Department of Archaeology, University of Durham, South Road, Durham, DH1 3LE UK; 4 Department of Biology, Saint Mary’s College of California, 1928 Saint Mary’s Road, Moraga, CA 94575 USA; 5 Department of Cultures, Archaeology, University of Helsinki, PO Box 59, 00014 Helsinki, Finland; 6 Archaeology Department, Institute of History and Archaeology, University of Tartu, Jakobi 2, 5101 Tartu, Estonia; 7 Department of Geosciences and Geography, University of Helsinki, PO Box 64, 00014 Helsinki, Finland; 8 EPOC, UMR 5805, University of Bordeaux, 33615 Pessac, France; 9 Faculty of Medicine, University of Helsinki, PO Box 63, 00014 Helsinki, Finland

**Keywords:** Archaeology, Palaeontology

## Abstract

Dogs (*Canis familiaris*) are the first animals to be domesticated by humans and the only ones domesticated by mobile hunter-gatherers. Wolves and humans were both persistent, pack hunters of large prey. They were species competing over resources in partially overlapping ecological niches and capable of killing each other. How could humans possibly have domesticated a competitive species? Here we present a new hypothesis based on food/resource partitioning between humans and incipient domesticated wolves/dogs. Humans are not fully adapted to a carnivorous diet; human consumption of meat is limited by the liver’s capacity to metabolize protein. Contrary to humans, wolves can thrive on lean meat for months. We present here data showing that all the Pleistocene archeological sites with dog or incipient dog remains are from areas that were analogous to subarctic and arctic environments. Our calculations show that during harsh winters, when game is lean and devoid of fat, Late Pleistocene hunters-gatherers in Eurasia would have a surplus of animal derived protein that could have been shared with incipient dogs. Our partitioning theory explains how competition may have been ameliorated during the initial phase of dog domestication. Following this initial period, incipient dogs would have become docile, being utilized in a multitude of ways such as hunting companions, beasts of burden and guards as well as going through many similar evolutionary changes as humans.

## Introduction

Humans and wolves belong to the highly competitive large carnivore guild^1,2^. When resources/game are abundant, different species of carnivores may tolerate each other in a sympatric relationship in which top carnivores provide carcasses for other guild members to scavenge. During lean times, direct and indirect negative interactions between guild members predominate^3^. Accordingly, it would be highly likely that prehistoric hunter-gatherers would have killed wolves as ecological competitors rather than tolerated them.

Humans are unusual carnivores, in that we are primates with ancestors that were herbivores and insectivores, and at the same time prey to larger carnivores. During the Pliocene– Pleistocene transition, some early hominins adapted to scavenging as an important part of foraging activities^4,5^. When the larger, large-brained *Homo* genus appeared, the hominin clade entered the carnivore guild^3^. This atypical evolutionary history means that humans have an incomplete ability to digest meat and must rely on exosomatic adaptations to hunt large game. Wolves on the contrary are typical carnivores.

The specifics of early dog domestication are uncertain. Palaeolithic humans are known to have engaged in interaction with the predatory species^6^. Carnivores are found at Palaeolithic sites with cut marks suggesting ritual butchery such as brain removal, and there is evidence of wolf burials at Upper Palaeolithic sites^7,8^. The first signs of proto-dogs appear in Upper Palaeolithic deposits in Eurasia (map 1, Supplementary Materials). Genetic studies suggest that dogs descend from extinct wolf populations that diverged from the ancestors of extant wolves approximately 27,000–40,000 years ago^6^. The wolf population that dogs most likely descend from was large Northern wolves^9,10^. Moreover, rather than being a single event, domestication appears to be a complex process with dogs continuing to interbreed with wild wolves^11,12^.

There is little doubt that similarities between human and wolf societies facilitated in the process of wolf domestication. Following the initial phase of domestication, a process of coevolution appears to have taken place, which explains some traits shared by humans and dogs^13^. The domestication of dogs has increased the success of both species to the point that dogs are now the most numerous carnivore on the planet^9^. How this mutually beneficial relationship emerged, and specifically how the potentially fierce competition between these two carnivores was ameliorated, needs to be explained.

## The arctic setting: the area of early domestication

**Figure 1:**
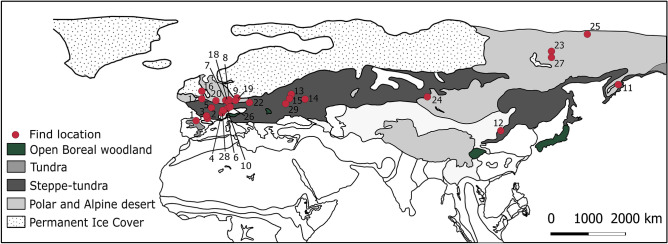
A map of vegetation zones during the Last Glacial Maximum^40^ and Palaeolithic dog remain discoveries (see supplementary).

The initial wolf domestication fell within the comparatively short glacial maximum at the latter part of the ice age (Last Glacial Maximum (LGM), corresponding with Marine Isotope Stage 2; 14–29 kyr, see Fig. 1), when the global ice volume reached its maximum. During the LGM cold snap, fossil evidence suggests continuous tundra/tundra-steppe environments in central European sites, which had remained wooded for much of the preceding ice age. Although there is still discussion of microrefugia in western Europe during the LGM, most studies support the view that western Europe was predominantly covered by a steppe-tundra biome^14–16^. Glaciers covered much of the western part of Eurasia^17^ (see Fig. 1).

Recent assessments of the available palaeoclimate data have emphasized the highly continental nature of the ice age climate with severely cold winters in the ice-free land areas of northern Eurasia^18^. Based on syntheses of fossil data and numerical palaeoclimate modelling for the LGM, there is strong evidence that there was a significant fall in annual temperatures compared to the present across northern Eurasia, but that there may have been only moderate reductions in summer temperature, in some regions as small as 1–2 °C (but commonly in the 4–8 °C range), while the fall in winter temperature is considerably larger, typically 10 °C or more^19,20^.

## Results and discussion

Human populations in Eurasia during the LGM would have relied on an animal-based diet during the exceptionally harsh winters. The availability of plant-based products (the majority of carbohydrates in the diet) is limited by a short growing season. Plant products can be stored, but these resources significantly diminish in late winter^21^. This is seen today in northern latitudes, where people consume higher levels of animal derived calories^22,23^. This leads to a diet with plentiful protein but limited resources of fat or carbohydrates.

Humans are not adapted to a solely carnivorous diet and are only able to digest about 20% of their energy needs from protein^24^. High consumption of protein may lead to hyperinsulinemia, hyperammonia or diarrhea. In the worst case excessive lean meat consumption may lead to fatal protein poisoning^22^.

Contrary to warmer environments where access to protein can be a limiting factor for human population size, in arctic and subarctic environments, human fecundity and survival is dependent on carbohydrates and/or fat availability^25^. Modern human populations have means to avoid excessive amounts of animal protein in their diet by shifting their exploitation strategies toward fauna retaining higher levels of fat deposits such as fish and bear and avoiding lean meat^21^. However, animal derived protein can seasonally account for as much as 45% of the caloric intake of arctic hunter-gatherers^26^.

During harsh winters, meat would not always have been a favorable food. Hunter-gatherers can avoid lean meat by modifying how they butcher ungulates in order to focus on body elements from which fat and grease can be extracted. Distal limbs and crania maintain fat deposits^27^. Limb bones can be used to extract fatty oils, and there is evidence for such processing behavior during the Upper Palaeolithic^28–30^.

**Figure 2:**
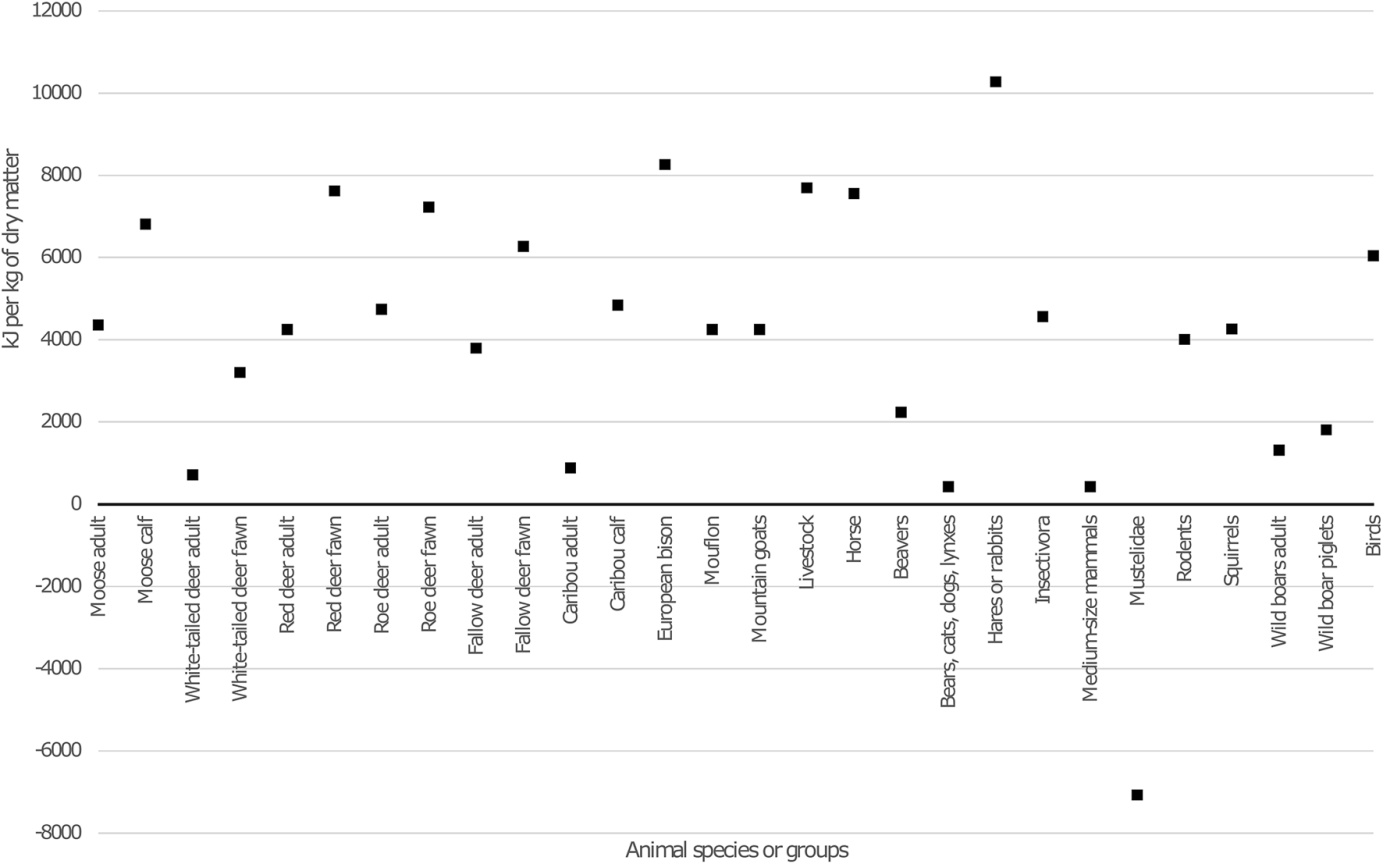
Humans have a limited capacity to digest protein. Calculation based on 45% of energy demands from animal protein leads to “left over” energy of hundreds or thousands kJ per kg of dry animal (see supplementary). This excess protein can be fed to proto-dogs.

Unlike humans, wolves can, because of their evolutionary history as carnivores, sustain in the short-term on a solely protein-based diet^31^. To test if humans and wolves could co-exist without competition over resources, we calculated the left over energy for the main prey available to wolves during the Late Pleistocene and Early Holocene (see Fig. 2 and supplementary). These calculations are likely to be conservative because the lipid composition of game varies between seasons and are at the lowest during critical winter months. We have used mean estimations for fat content and overestimation on ability to digest protein, showing that even with a higher lipid content, our hypothesis still works.

Our calculations show that (see Fig. 2), apart from Mustelidae, every one of the prey species of wolves have protein ratios over the limit that humans can consume. This ‘overproduction’ of protein in arctic and subarctic environments could easily have been fed to wolves/dogs when kept as a pet. Therefore, in the short term over the critical winter months, wolves and humans would not have been in competition over resources and may have mutually benefited from each other’s companionship. This would have been critical in keeping the first proto-dogs for years and generations.

Hunter-gatherers are known to take pets, thus the idea that Palaeolithic people captured wolf pups for pets is reasonable^32^. Keeping such a pet over several generations would only be feasible if there were enough year-round caloric resources for both humans and pets alike over several generations. Given this precondition, most animals kept by hunter-gatherers never become tame. According to our theory, late Pleistocene hunter-gatherers in Eurasia would have had enough free/excess animal derived calories to feed proto-dogs/captured wolves during lean winter months and therefore humans and canids would not have been in competition over resources. Given that there would not have been competition over resources, even a small benefit from keeping captive wolves, such as hunting aid or protection against predators, would have been advantageous for both species. This also explains how several independent domestication events may have taken place across Eurasia during the Last Glacial^11,12^.

The question of why dogs were domesticated has been long debated without any conclusive arguments. Domesticating wolves was a new form of positive interaction within the carnivore guild. This process has been explained by two hypotheses: one posits that humans actively tamed dogs as hunting partners; the other argues that docile wolves (short fleeing distance) were attracted to waste zones near human settlements and gradually adapted to life alongside humans^33^. Both hypotheses are problematic.

It is unlikely that untamed wolves were cooperative hunting partners with humans. Moreover, the dogs do not necessary increase hunting success^34^. Tamed wolves would only have been significant hunting partners after collaboration and highly advanced communication between the two species evolved. This is not likely to have happened during the initial pet stage but later when dogs were domesticated and sociable with humans. The hunting partner theory does not work in cold regions because dogs are likely to increase hunting success only outside of the natural habitat of grey wolves, thus not in the area of initial domestication^34^. Although the Palaeolithic wolf habitat is unknown, it very likely covered the arctic or subarctic regions, thus making the actual domestication region poorly compatible with the ‘hunting partner’ model.

It is also unlikely that wolves were attracted to human waste. During the Palaeolithic, it is unlikely that humans occupied sedentary or semi-sedentary sites where substantial amounts of waste could be generated^35^. Moreover, based on isotopic analysis, early dogs had a different diet from humans^36,37^ suggesting that early dogs were not adapted to consuming human food waste but were selectively fed a terrestrial animal based diet—in our hypothesis, surplus protein. In this way our partitioning theory supports the hypothesis that human food waste played a role in dog domestication, although not necessarily discarded foods, but human excess lean meat which may have been actively fed to dogs.

We suggest that the domestication of dog needs to be understood in terms of competition over resources in the particularly severe environment that prevailed in northern Eurasia during the latter part of the Last Ice Age. In such a context, Upper Palaeolithic hunter-gatherers would have had excess protein available to feed to captured/pet wolves. The digestive system of early dogs was not adapted to a diet high in floral resources^31^.

Currently most modern dogs have an expansion of genes coding for amylase, indicating an increased ability to digest starch^38^. This expansion is lacking in some ancient breeds and the copy number vary even in wolves, suggesting that this was not a shared feature in early dogs. Contrary to later periods where dogs are shown to eat a largely similar diet to humans^39^, the diet of Palaeolithic dogs probably mostly consisted of only terrestrial meat^36^.

## Conclusions

We suggest that the differences between dietary constraints of wolves and humans enabled dog domestication in harsh environments in the Late Pleistocene. Excess protein decreased dietary competition and enhanced the possibility of sympatric existence. This could have been a significant impetus for wolves to become “our best friend”.

## Methods

A list of the main prey species of wolves are included in the supplemental materials. The energy content was calculated according to the Atwaters system, being 17 MJ/kg for protein and 38 MJ/kg for lipids and it was based on the total energy content of each animal.

The energy per kg of average animal (dry weight) left over when 45% of caloric intake would be protein was calculated as:

$${\text{Energy left over from protein }} = \, \left( {{\text{CP}}*{17}} \right) - \left( {{\text{EE}}*{38}*{45}/{55}} \right)$$

where CP = Crude protein (g/100 g DM), EE = Ether Extract (g/100 g DM).

## Supplementary information

Supplementary Information.

## Data Availability

All data generated or analysed during this study are included in this published article and its Supplementary Information files.
