# Supplementary material for: Excess protein enabled dog domestication during severe Ice Age winters
Source: Sci Rep. 2021 Jan 7;11:7. doi: 10.1038/s41598-020-78214-4 (PMC7790815; doi:10.1038/s41598-020-78214-4)
Supplement: Supplementary file 1 — Supplementary Information. [file 41598_2020_78214_MOESM1_ESM.docx]

Supplementary information (S1) for

Excess protein enabled dog domestication during severe Ice Age winters

**Maria Lahtinen*^1,2^, David Clinnick^3,4^, Kristiina Mannermaa^5,6^, J. Sakari Salonen^7,8^, Suvi Viranta^9^**

1. **Finnish Food Authority, Mustialankatu 3, 00790 Helsinki, Finland**
2. **University of Helsinki, Finnish Museum of Natural History, Laboratory of Chronology, PO Box 64, 00014 University of Helsinki, Finland**
3. **University of Durham, Department of Archaeology, South Road, DH1 3LE, Durham, UK**
4. **Saint Mary's College of California, Department of Biology, 1928 Saint Mary's Road, Moraga, CA 94575, USA**
5. **Tartu University, University of Tartu, Archaeology Department, Institute of History and Archaeology, University of Tartu, Jakobi 2, 5101 Tartu, Estonia**
6. **University of Helsinki, Department of Cultures, Archaeology, PO Box 59, 00014 University of Helsinki, Finland**
7. **University of Helsinki, Department of Geosciences and Geography, PO Box 64, 00014 University of Helsinki, Finland**
8. **University of Bordeaux, EPOC, UMR 5805, F-33615 Pessac, France**
9. **University of Helsinki, Faculty of Medicine, PO Box 63, 00014 University of Helsinki, Finland**

***corresponding author** [**maria.lahtinen@finnishfoodauthority.fi**](mailto:maria.lahtinen@finnishfoodauthority.fi)

| TABLE 1 |  |  |  |  |  | 45% protein in diet |
| --- | --- | --- | --- | --- | --- | --- |
|  | Scientific name | DM | CP | EE | Reference | kJ of kg DM |
| Ungulates |  | % | (g/100g DM) | (g/100g DM) |  |  |
| Moose adult | *Alces alces* | 38,90 | 69,90 | 24,20 | (*1*) | 4359 |
| Moose calf | *Alces alces* | 37,00 | 73,00 | 18,00 | (*1*) | 6814 |
| White-tailed deer adult | *Odocoileus virginianus* | 40,90 | 62,40 | 31,80 | (*1*) | 721 |
| White-tailed deer fawn | *Odocoileus virginianus* | 39,10 | 65,50 | 25,50 | (*1*) | 3207 |
| Red deer adult | *Cervus elaphus* | 39,00 | 68,90 | 24,00 | (*1*) | 4251 |
| Red deer fawn | *Cervus elaphus* | 38,70 | 72,10 | 14,90 | (*1*) | 7624 |
| Roe deer adult | *Capreolus capreolus* | 38,30 | 70,50 | 23,30 | (*1*) | 4741 |
| Roe deer fawn | *Capreolus capreolus* | 36,50 | 73,60 | 17,00 | (*1*) | 7227 |
| Fallow deer adult | *Dama dama* | 38,20 | 68,60 | 25,30 | (*1*) | 3796 |
| Fallow deer fawn | *Dama dama* | 36,30 | 71,80 | 19,10 | (*1*) | 6268 |
| Caribou adult | *Rangifer tarandus* | 44,10 | 62,10 | 31,10 | (*1*) | 888 |
| Caribou calf | *Rangifer tarandus* | 43,00 | 65,80 | 20,40 | (*1*) | 4844 |
| European bison | *Bison bonasus* | 32,50 | 76,40 | 15,20 | (*1*) | 8262 |
| Mouflon | *Ovis aries orientalis* | 39,00 | 68,90 | 24,00 | (*1*) | 4251 |
| Mountain goats | *Oreamnos americanus* | 39,00 | 68,90 | 24,00 | (*1*) | 4251 |
| Livestock |  | 32,20 | 74,90 | 16,20 | (*1*) | 7696 |
| Horse | *Equus cabellus* |  | 79,02 | 18,90 | (*2*) | 7557 |
| Non-ungulates |  |  |  |  |  |  |
| Beavers | *Castor* | 35,50 | 58,50 | 24,80 | (*1*) | 2234 |
| Bears, cats, dogs, lynxes |  | 38,80 | 55,20 | 28,80 | (*1*) | 430 |
| Hares or rabbits | *Leporidae* | 31,90 | 71,80 | 6,20 | (*3*–*6*) | 10278 |
| Insectivora | *insectum* | 31,20 | 61,60 | 19,00 | (*3*) | 4565 |
| FoxIEs | *Vulpes* | 38,80 | 55,20 | 28,80 | (*1*, *7*, *8*) | 430 |
| Mustelidae | *mustela* | 38,10 | 39,60 | 44,40 | (*1*) | -7072 |
| Rodents | *Rodere* | 32,10 | 61,80 | 20,90 | (*3*) | 4008 |
| Squirrels | *Sciuridae* | 31,10 | 65,50 | 22,10 | (*3*) | 4264 |
| Wild boars adult | *Sus scrofa* | 42,50 | 62,80 | 30,10 | (*1*) | 1318 |
| Wild boar piglets | *Sus scrofa* | 23,20 | 54,90 | 24,20 | (*1*) | 1809 |
| Birds | *Aves* | 31,60 | 64,60 | 15,90 | (*3*) | 6039 |

**TABLE 2 Location of the earliest dog/protodog discoveries, dates and references**

|  |  |  |  |  |  |  |
| --- | --- | --- | --- | --- | --- | --- |
| Location on the map | Current Country | Site | Date (BP) | Date (cal BP) | Dog reference | Dating Reference |
|  |  |  |  |  |  |  |
| 16 | United Kingdom | Star Carr | 9559+-210 | 11,658 - 10,633 | (*9*, *10*) | (*9*) |
| 17 | United Kingdom | Seamer Carr | 9940+-100 | 11,866 - 11,246 | (*9*) | (*9*) |
| 26 | Germany | Hohle Fels |  | 40,000-29,000 | (*11*) | (*12*) |
| 7 | Germany | Bonn-Oberkassel | 12,270+-100; 12200+-60; 12100+-45; 11620+-60 | 14,708 - 13,874 | (*13*) | (*13*, *14*) |
| 9 | Germany | Oelknitz |  | 15,145 - 12,915 | (*15*) | (*16*) |
| 19 | Germany | Döbritz, Kniegortte |  | 16,900 - 11,760 | (*15*) | (*16*) |
| 18 | Germany | Teufelsbrucke |  | 15,877 – 13,986 | (*15*) | (*16*) |
| 8 | Germany | Senckenberg |  | Late Paleolithic or Early Mesolithic | (*10*) | (*10*) |
| 10 | Switzerland | Kesslerloch Cave | 12.225±45 | 12,334 - 12,027 | (*17*) | (*17*) |
| 28 | Switzerland | Monruz |  | 16,345 – 14,952 | (*18*) | (*14*) |
| 6 | Switzerland | Hauterive-Champréveyres |  | c. 15-14,000 | (*19*) | (*20*) |
| 4 | France | Saint-Thibaud-de-Couz | 10,050± 100 | 12,027 - 11,311 | (*21*) | (*22*) |
| 3 | France | Pont d'Ambon | 10730+-100 | 12,952 - 12,451 | (*22*) | (*23*) |
| 2 | France | Montespan |  | 15,500 - 13,500 | (*22*) | (*24*) |
| 5 | France | Le Closeau |  | 14,999 - 14,055 | (*22*) | (*22*) |
| 1 | Spain | Erralla |  | between 20,000 and 12,500 | (*25*) | (*25*, *26*) |
| 13 | Russia | Eliseevichi I | 13905+-55 | 15,124 - 14,637 | (*27*) | (*28*) |
| 15 | Ukraine | Mezin |  | 14,700 – 14,300 | (*29*) | (*22*) |
| 29 | Ukraine | Mezhirich |  | 15,000 - 14,000 | (*29*) | (*30*) |
| 22 | Czech Republic | Gravettian Předmostí | 24,492 ± 67 | 28,754 - 28,328 | (*31*) | (*32*) |
| 20 | Belgium | Goyet | 31,680 ± 250 | 34,999 - 36,128 | (*29*) | (*29*) |
| 27 | Russia | Ullakhan Sular | 13925 ± 70 | 15,191- 14,635 | (*33*) | (*33*) |
| 23 | Russia | Yana RHS, Lower Yana River | 28 520+-240/28 840+-220 | 33,000–33,500 | (*34*) | (*34*) |
| 14 | Russia | Kostenki 8 |  | approx. 33,500 - 22,000 | (*35*) | (*35*) |
| 24 | Russia | Razboinichya Cave | 29915±1000 | 33,826 - 29,640 | (*36*) | (*33*) |
| 25 | Russia | Zhokhov site | 8710 ± 50; 8910 ± 50 | 9,557 -10,203 | (*37*) | (*37*) |
| 11 | Russia | Ushki I |  | c. 13,000 | (*38*) | (*38*, *39*) |
| 12 | China | Nanzhuangtou |  | 12,000 – 10,500 | (*40*) | (*41*) |

1. G. Bosch, E. A. Hagen-Plantinga, W. H. Hendriks, Dietary nutrient profiles of wild wolves: insights for optimal dog nutrition? *Br. J. Nutr.* **113**, S40–S54 (2015).

2. J. Robb, R. B. Harper, H. F. Hintz, J. T. Reid, J. E. Lowe, H. F. Schryver, M. S. S. Rhee, Chemical composition and energy value of the body, fatty acid composition of adipose tissue, and liver and kidney size in the horse. *Anim. Sci.* **14**, 25–34 (1972).

3. E. A. Plantinga, G. Bosch, W. H. Hendriks, Estimation of the dietary nutrient profile of free-roaming feral cats: possible implications for nutrition of domestic cats. *Br. J. Nutr.* **106**, S35–S48 (2011).

4. J. A. Litvaitis, W. W. Mautz, Food and Energy Use by Captive Coyotes. *J. Wildl. Manag.* **44**, 56–61 (1980).

5. J. G. Powers, W. W. Mautz, P. J. Pekins, Nutrient and Energy Assimilation of Prey by Bobcats. *J. Wildl. Manag.* **53**, 1004–1008 (1989).

6. R. P. Davison, W. W. Mautz, H. H. Hayes, J. B. Holter, The Efficiency of Food Utilization and Energy Requirements of Captive Female Fishers. *J. Wildl. Manag.* **42**, 811–821 (1978).

7. C. Lefebvre, M. Crête, J. Huot, R. Patenaude, Prediction of body composition of live and post-mortem red foxes. *J. Wildl. Dis.* **35**, 161–170 (1999).

8. P. Prestrud, K. Nilssen, Fat Deposition and Seasonal Variation in Body Composition of Arctic Foxes in Svalbard. *J. Wildl. Manag.* **56**, 221–233 (1992).

9. J. Clutton-Brock, N. Noe-Nygaard, New osteological and C-isotope evidence on mesolithic dogs: Companions to hunters and fishers at Star Carr, Seamer Carr and Kongemose. *J. Archaeol. Sci.* **17**, 643–653 (1990).

10. M. Degerbøl, On a find of a Preboreal domestic dog (Canis familiaris) from Star Carr, Yorkshire, with remarks on other Mesolithic dogs. *Proc. Prehist. Soc.* **27**, 35–55 (1961).

11. E. Camarós, S. C. Münzel, M. Cueto, F. Rivals, N. J. Conard, The evolution of Paleolithic hominin–carnivore interaction written in teeth: Stories from the Swabian Jura (Germany). *J. Archaeol. Sci. Rep.* **6**, 798–809 (2016).

12. N. J. Conard, M. Bolus, Radiocarbon dating the appearance of modern humans andtiming of cultural innovations in Europe: new results andnew challenges. *Jounal Hum. Evol.* **2003**, 331–371 (2003).

13. M. Street, Ein Wiedersehen mit dem Hund von Bonn-Oberkassel. *Bonn. Zool. Beitr.* **50**, 269–290 (2002).

14. M. Street, H. Napierala, L. Janssens, The late Palaeolithic dog from Bonn-Oberkassel in context. *Late Glacial Burial Oberkassel Revisit.*, 253–274 (2015).

15. R. Musil, in *Dogs Through Time: An Archaeological Perspective* (2002), *BAR*.

16. R. Feustel, MAG DALENI ENSTA TlON TEUFELSBROCKE I I: Archäologischer Teil. *Weimar. Monogr. ZUR UR- FROHGESCHICHTE* (1980).

17. H. Napierala, H.-P. Uerpmann, A ‘new’ palaeolithic dog from central Europe. *Int. J. Osteoarchaeol.* **22**, 127–137 (2012).

18. W. Müller, Le site magdalenien de Monruz, 3 Acquisition, traitement et consommation des ressources animales. *Archeol. Neuchateloise*. **49**, 14 (2013).

19. P. Morel, W. Müller, in *Archéologie neuchâteloise* (2004), pp. 96–109.

20. D. Leesch, M.-I. Cattin, W. Müller, Témoins d’implantations magdaléniennes et aziliennes sur la rive nord du lac de Neuchâtel. *Archéologie Neuchâtel.*, 13 (2009).

21. L. Chaix, A preboreal dog from the northern alps (Savoie, France). *Dogs Trough Time Archaeol. Perspect.* **889**, 49–59 (2000).

22. M. Pionnier-Capitan, C. Bemilli, P. Bodu, G. Célérier, J.-G. Ferrié, P. Fosse, M. Garcià, J.-D. Vigne, New evidence for Upper Palaeolithic small domestic dogs in South-Western Europe. *J. Archaeol. Sci.* **38**, 2123–2140 (2011).

23. G. Célérier, N. Tisnerat, H. Valladas, Données nouvelles sur l’âge des vestiges de chien à Pont d’Ambon, Bourdeilles (Dordogne)/New data on the age of Canis remains at Pont d’Ambon, Bourdeilles (Dordogne, France). *Paléo Rev. Archéologie Préhistorique*. **11**, 163–165 (1999).

24. M. A. Garcià, Montespan : modelages et remodelage. *Bull. Société Préhistorique Fr.* **86**, 259–260 (1989).

25. J. Altuna, K. Mariezkurrena, Bases de subsistencia de los pobladores de Erralla: Macromamíferos. *Munibe*, 87–117 (1985).

26. J.-D. Vigne, L’humérus de chien magdalénien de Erralla (Gipuzkoa, Espagne) et la domestication tardiglaciaire du loup en Europe. *Munibe*, 279–287 (2005).

27. M. V. Sablin, G. A. Khlopachev, The Earliest Ice Age Dogs: Evidence from Eliseevichi 1. *Curr. Anthropol.* **43**, 795–799 (2002).

28. M. V. Sablin, G. A. Khlopachev, SablinKhDie ältesten Hunde aus Eliseevici I (Russland). *Archäol. Korresp.*, 309–316 (2003).

29. M. Germonpré, M. V. Sablin, R. E. Stevens, R. E. M. Hedges, M. Hofreiter, M. Stiller, V. R. Després, Fossil dogs and wolves from Palaeolithic sites in Belgium, the Ukraine and Russia: osteometry, ancient DNA and stable isotopes. *J. Archaeol. Sci.* **36**, 473–490 (2009).

30. L. Marquer, V. Lebreton, T. Otto, H. Valladas, P. Haesaerts, E. Messager, D. Nuzhnyi, S. Péan, Charcoal scarcity in Epigravettian settlements with mammoth bone dwellings: the taphonomic evidence from Mezhyrich (Ukraine). *J. Archaeol. Sci.* **39**, 109–120 (2012).

31. M. Germonpré, M. Lázničková-Galetová, M. V. Sablin, Palaeolithic dog skulls at the Gravettian Předmostí site, the Czech Republic. *J. Archaeol. Sci.* **39**, 184–202 (2012).

32. M. Germonpré, M. Lázničková-Galetová, E.-L. Jimenez, R. Losey, M. Sablin, H. Bocherens, M. Van Den Broeck, Consumption of canid meat at the Gravettian Předmostí site, the Czech Republic. *Foss. Impr.* **73**, 360–382 (2017).

33. M. Germonpré, S. Fedorov, P. Danilov, P. Galeta, E.-L. Jimenez, M. Sablin, R. J. Losey, Palaeolithic and prehistoric dogs and Pleistocene wolves from Yakutia: Identification of isolated skulls. *J. Archaeol. Sci.* **78**, 1–19 (2017).

34. E. J. Lee, D. A. Merriwether, A. K. Kasparov, P. A. Nikolskiy, M. V. Sotnikova, E. Y. Pavlova, V. V. Pitulko, Ancient DNA Analysis of the Oldest Canid Species from the Siberian Arctic and Genetic Contribution to the Domestic Dog. *PLOS ONE*. **10**, e0125759 (2015).

35. M. Germonpré, M. Lázničková-Galetová, R. J. Losey, J. Räikkönen, M. V. Sablin, Large canids at the Gravettian Předmostí site, the Czech Republic: The mandible. *Quat. Int.* **359–360**, 261–279 (2015).

36. N. D. Ovodov, S. J. Crockford, Y. V. Kuzmin, T. F. G. Higham, G. W. L. Hodgins, J. van der Plicht, A 33,000-Year-Old Incipient Dog from the Altai Mountains of Siberia: Evidence of the Earliest Domestication Disrupted by the Last Glacial Maximum. *PLoS ONE*. **6**, e22821 (2011).

37. V. V. Pitulko, A. K. Kasparov, Archaeological dogs from the Early Holocene Zhokhov site in the Eastern Siberian Arctic. *J. Archaeol. Sci. Rep.* **13**, 491–515 (2017).

38. N. D. Dikov, in *Amaricas Beginnings: The Prehistory and Paleoecology of Beringia* (1996), pp. 244–250.

39. T. Goebel, S. B. Slobodin, M. R. Waters, New dates from Ushki-1, Kamchatka, confirm 13,000calBP age for earliest Paleolithic occupation. *J. Archaeol. Sci.* **37**, 2640–2649 (2010).

40. Y. Jing, Zhongguo gu dai jia yang dong wu de dong wu kao gu xue yan jiu (ZOOARCHAEOLOGICAL STUDY ON THE DOMESTIC ANIMALS IN ANCIENT CHINA). *Quoternary Sci.* **30**, 385–391 (2010).

41. D. J. Cohen, The Beginnings of Agriculture in China: A Multiregional View. *Curr. Anthropol.* **52**, S273–S293 (2011).
